# Synergistic Effects of Artesunate in Combination with Amphotericin B and Miltefosine against *Leishmania infantum*: Potential for Dose Reduction and Enhanced Therapeutic Strategies

**DOI:** 10.3390/antibiotics13090806

**Published:** 2024-08-26

**Authors:** Nuchpicha Intakhan, Atiporn Saeung, Sonia M. Rodrigues Oliveira, Maria de Lourdes Pereira, Wetpisit Chanmol

**Affiliations:** 1 School of Allied Health Sciences, Walailak University, Nakhon Si Thammarat 80160, Thailand; nuchpicha.in@wu.ac.th; 2 Center of Excellence Research for Melioidosis and Microorganisms, School of Allied Health Sciences, Walailak University, Nakhon Si Thammarat 80160, Thailand; 3 Department of Parasitology, Faculty of Medicine, Chiang Mai University, Chiang Mai 50200, Thailand; atisaeung.noi@gmail.com; 4 CICECO-Aveiro Institute of Materials, University of Aveiro, 3810-193 Aveiro, Portugal; sonia.oliveira@ua.pt (S.M.R.O.); mlourdespereira@ua.pt (M.d.L.P.); 5 HMRI—Hunter Medical Research Institute, New Lambton, NSW 2305, Australia; 6 Department of Medical Sciences, University of Aveiro, 3810-193 Aveiro, Portugal

**Keywords:** leishmaniasis, antiparasitic agents, drug combinations, artesunate, drug therapy, zoonosis

## Abstract

Leishmaniasis is a tropical infectious disease caused by *Leishmania* parasites. The disease can be spread by the bite of an infected sand fly. Currently, five chemotherapeutic drugs are available in leishmaniasis treatment. However, these drugs exhibit toxicity and serious adverse effects on infected individuals, necessitating alternative treatment strategies. One such strategy involves using combinations of existing antileishmanial drugs. In this study, we evaluated the interaction between artesunate (AS) and three antileishmanial drugs—amphotericin B (AmB), miltefosine (MF), and paromomycin (PM) against *Leishmania infantum*. This evaluation marks the first time such an assessment has been conducted. The Chou–Talalay combination index method was employed to analyze the drug interaction. The findings revealed that the interaction between AS and AmB ranged from antagonistic to synergistic, while the interaction between AS and MF showed moderate to strong synergism. In contrast, the interaction between AS and PM resulted in an antagonistic interaction, which differs from the combinations with AmB or MF. This study provides valuable insights for developing novel drug regimens for leishmaniasis treatment, emphasizing the potential of AS and its combination with existing antileishmanial drugs. Further research is necessary to optimize drug combinations and minimize adverse effects, leading to more effective therapeutic outcomes.

## 1. Introduction

Leishmaniasis is a neglected tropical disease that can be spread by the bite of an infected sand fly [1]. This disease is prevalent in 98 countries, primarily in tropical and subtropical regions, with approximately 1 million new cases reported each year and an estimated 1 billion people at risk worldwide [2]. Leishmaniasis manifests across a broad clinical spectrum, ranging from asymptomatic, self-resolving, localized skin lesions to progressive forms such as cutaneous (CL), mucocutaneous (MCL) and, the most severe form, visceral leishmaniasis (VL) [3]. If left untreated, VL can be fatal [4]. VL is a chronic systemic disease characterized by symptoms such as fever, weight loss, hepatosplenomegaly, and severe anemia or pancytopenia [5]. The primary causative agents of VL are *L*. *donovani* and *L. infantum*. Leishmaniasis is categorized into two types based on the nature of their reservoir hosts—zoonotic and anthroponotic. The life cycle of the *Leishmania* parasite includes an extracellular promastigote stage in the gut of the phlebotomine sand fly and an intracellular amastigote stage in the vertebrate host [6]. Inside the gut of the sand fly, the parasite exists in various flagellated forms to adapt and survive. As the parasites proliferate and mature into metacyclic promastigotes, they detach from the midgut epithelium and are transmitted to a vertebrate host during the sand fly’s next blood meal. Once inside the vertebrate host, the metacyclic promastigotes transform into non-motile amastigotes, which then persist within the phagosomes of host cells [7]. A significant proportion of *Leishmania* species are implicated in zoonotic transmission. *L. infantum* (syn. *chagasi*) is particularly involved in zoonotic transmission and is a significant cause of global leishmaniasis. This species is known to cause human VL and canine leishmaniasis (CanL) [8]. Domestic dogs play a significant role in *Leishmania* transmission due to their proximity to humans and high susceptibility to the parasite. Infected dogs serve as accessible reservoirs for sand flies, facilitating the transmission of the parasite to humans and maintaining its presence in the environment [9].

Current treatments for leishmaniasis primarily involve the following five chemotherapeutic drugs: pentavalent antimonials (SbV), Amphotericin B (AmB), Miltefosine (MF), Paromomycin (PM), and Pentamidine (PEN). However, these drugs often exhibit toxicity and adverse effects in patients [10]. Pentavalent antimonials have been the first-line treatment for VL since 1945, with an effectiveness ranging from 35% to 95% depending on the region [11]. Nevertheless, its usage is increasingly limited due to significant toxicity, including cardiotoxicity, pancreatitis, hepatotoxicity, and nephrotoxicity [12], along with the emergence of drug-resistant parasites [11,13]. Amphotericin B deoxycholate (AmB) is a second-line treatment for VL, but it has limitations such as poor bioavailability, renal toxicity, and infusion-associated reactions like thrombophlebitis [14]. Its parenteral administration requires a lengthy hospitalization period [15]. Liposomal formulations of AmB (Ambisome^®^; Gilead Sciences, Inc., Foster City, CA, USA) provide fewer toxic alternatives but are more expensive. Miltefosine (MF), the only orally administered drug for VL, has shown high efficacy in clinical trials [16] but has drawbacks including a lengthy treatment course, potential teratogenicity, and risk of resistance due to a long half-life [17]. Moreover, the teratogenic effects of MF also limit its use in women of reproductive age. Pentamidine is usually given to cases unresponsive to antimonials [18]. However, it has high toxicity, including cardiotoxicity, reduced blood pressure, and irreversible insulin-dependent diabetes mellitus [19]. Paromomycin (PM) is effective against various protozoan parasites, including *Leishmania* spp. [20], with a high cure rate in clinical trials [21], but its use as a monotherapy is constrained by drug resistance. Other therapeutic options like imiquimod, sitamaquine, and azoles have also been explored but share similar issues with toxicity. Drug combinations have historically been used to treat diseases and reduce suffering. Using combination drugs with different mechanisms or modes of action can enhance the effectiveness of treatment. Synergism in drug combinations helps increase therapeutic efficacy, reduce the dosage while maintaining or enhancing efficacy to avoid toxicity, and slow down or reduce the development of drug resistance [22]. Thus, the development of new therapeutic strategies is urgently needed, including drug combinations, to reduce toxicity and improve treatment outcomes for leishmaniasis.

Artemisinin and its derivatives are sesquiterpene trioxanes [23] that have demonstrated efficacy against human parasitic infections such as malaria [24]. Artemisinin-based combination therapies are recommended by the World Health Organization (WHO) for the treatment of malaria and the prevention of drug resistance. Artesunate is one of the artemisinin derivatives which has been FDA-approved for the treatment of severe malaria due to its favorable properties and safety profile. It is safe, well tolerated, stable, and soluble. Additionally, it can be administered to infants, children, and pregnant women with minimal toxicity. The safety of artesunate has been tested in various animal species, including rodents, rabbits, dogs, and macaques, with no adverse health effects observed, even at doses of up to 100–150 mg/kg [25]. Artesunate exhibits various pharmacological properties such as anti-cancer, anti-inflammatory, and anti-virus [26]. In *Leishmania*, artesunate showed its activity both in vitro and in vivo in a murine visceral leishmaniasis model [27,28,29,30]. It exhibited minimal side effects, indicating a higher safety index of the drug [30,31]. Moreover, artesunate has been proven effective in treating canine visceral leishmaniasis in naturally infected dogs [32]. Combination therapy for leishmaniasis has been developed, such as paromomycin plus miltefosine, liposomal amphotericin B plus miltefosine, and sodium stibogluconate/meglumine antimoniate plus paromomycin [13], to improve treatment efficacy, reduce the duration of treatment and hospitalization, and prevent the emergence of drug-resistant parasites [33].

However, there have been no investigations into the interaction between artesunate and other available antileishmanial drugs. Therefore, in this study, we explored, for the first time, the combination effects of artesunate with some currently available antileishmanial drugs, including amphotericin B, miltefosine, and paromomycin, against *L. infantum* intracellular amastigotes in THP-1-derived macrophages. The results of the study offer valuable insights for developing novel drug regimens for leishmaniasis treatment using a combination therapy approach.

## 2. Results

### 2.1. Antileishmanial Activity of AS, AmB, MF, and PM against L. infantum

The results demonstrated that all four drugs exhibited inhibitory effects on both promastigote and amastigote viability. Concentration–response curves were generated for each drug against the promastigotes and the intracellular amastigotes using GraphPad Prism software version 9, as shown in Figure S1. The half maximal inhibitory concentrations (IC_50_) of AS, AmB, MF, and PM against the promastigotes were determined to be 121.43 ± 2.05 µM, 0.02 ± 0.002 µM, 2.95 ± 0.18 µM, and 114.93 ± 9.46 µM, respectively (Table 1). To assess the activity of the drugs against the intracellular amastigotes, a parasite rescue and transformation assay was performed. The IC_50_ values for AS, MF, and PM were relatively low, measuring at 5.88 ± 1.67 µM, 0.37 ± 0.11 µM, and 64.30 ± 2.37 µM, respectively. In contrast, AmB exhibited a relatively higher IC_50_ value of 0.14 ± 0.02 µM compared to its activity against promastigotes.

### 2.2. Cytotoxicity of AS, AmB, MF, and PM to THP-1-Derived Macrophages

Concentration–response curves of the cytotoxicity of AS, AmB, MF, and PM were generated as shown in Figure S1. The CC_50_ values, which represent the drug concentration required to induce 50% cell death, were determined for AS, AmB, and MF as 225.77 ± 21.71 µM, 1.20 ± 0.27 µM, and 51.12 ± 0.65 µM, respectively. On the other hand, PM demonstrated likely non-cytotoxicity to THP-1-derived macrophages, with a CC_50_ greater than 20,000 µM (Table 1). To assess the safety and efficacy of the drugs, the selectivity index (SI) values, calculated as the ratio of CC_50_ to IC_50_, were determined. The SI value greater than 10 indicates that the drug is effective and safe [34]. The SI values of AS, AmB, and MF were 38.40, 8.57, and 138.16, respectively (Table 1) However, the exact SI value for PM could not be calculated, but it was estimated to be greater than 311. These results indicate that AmB was the most toxic among the four drugs, with the SI < 10. 

### 2.3. Combination Effects of Artesunate and Three Drugs against Intracellular Amastigotes

In this study, the drug interaction between AS and the antileishmanial drugs, including AmB, MF, and PM, was investigated in the intracellular amastigotes of *L. infantum* using the parasite rescue and transformation assay. Three pairs of drug combinations, namely AS–AmB, AS–MF, and AS–PM, were generated using the checkerboard method. 

**Figure 1:**
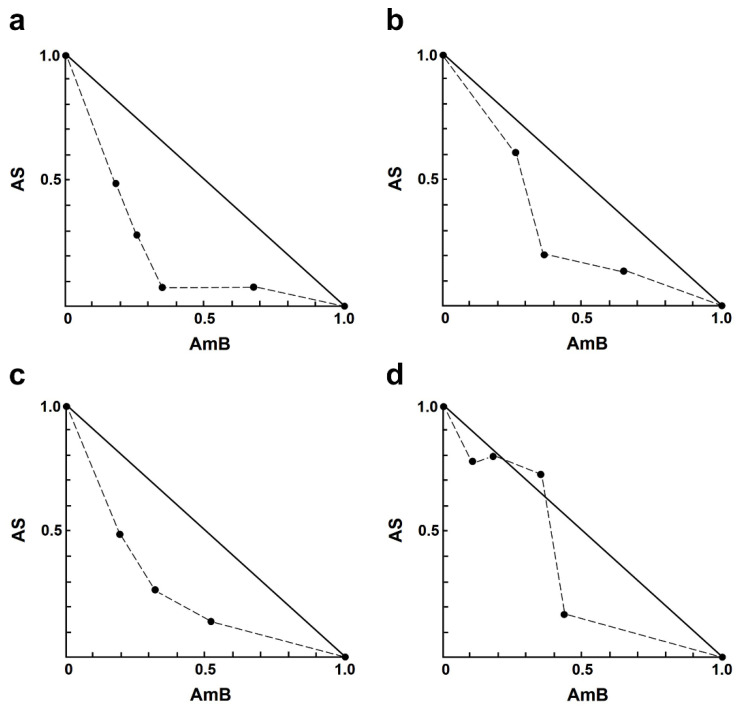
Representative normalized isobologram of the interaction between AS and AmB against *L. infantum* intracellular amastigotes. The *X*-and *Y*-axis represent dose-normalized isobolograms for AS and AmB, normalization of the dose with IC50 to unity on both axes. (**a**) 1.0 µM AS plus 0.03, 0.05, 0.10, and 0.20 µM AmB. (**b**) 2.0 µM AS plus 0.05, 0.10, and 0.20 µM AmB. (**c**) 4.0 µM AS plus 0.05, 0.10, and 0.20 µM AmB. (**d**) 8.0 µM AS plus 0.03, 0.05, 0.10, and 0.20 µM AmB.

For the AS–AmB combination, a concentration of AS at 8.0 µM and AmB at 0.20 µM resulted in approximately 83% and 55% growth inhibition, respectively. Sixteen different concentrations of drug combinations (AS–AmB) were tested on the intracellular amastigotes, and the percentage of growth inhibition was used to determine the combination index (CI) value for each combination, which indicates the type of drug interaction. The interaction between AS and AmB ranged from slight synergism to very strong antagonism (Table 2). Synergistic effects were observed for various combinations of AS (1.00–8.00 µM), including slight synergism in three concentrations, moderate synergism in one concentration, and synergism in eight concentrations (Table 2). However, there were two combinations that showed nearly additive 8.00 µM AS plus 0.05 or 0.1 µM AmB, while two concentrations showed strong antagonism 4.00 µM AS plus 0.03 µM AmB and very strong antagonism 2.00 µM AS plus 0.03 µM AmB. Isobologram analysis, representing the interaction between AS and AmB, indicated synergy for 12 combinations, with 12 data points falling below the line of additivity (Figure 1). Furthermore, combining 8.00 µM AS with 0.03 µM AmB resulted in the highest dose reduction index (DRI) value, indicating a significant reduction in the dose of AmB required, with a DRI value of 9.20.

**Figure 2:**
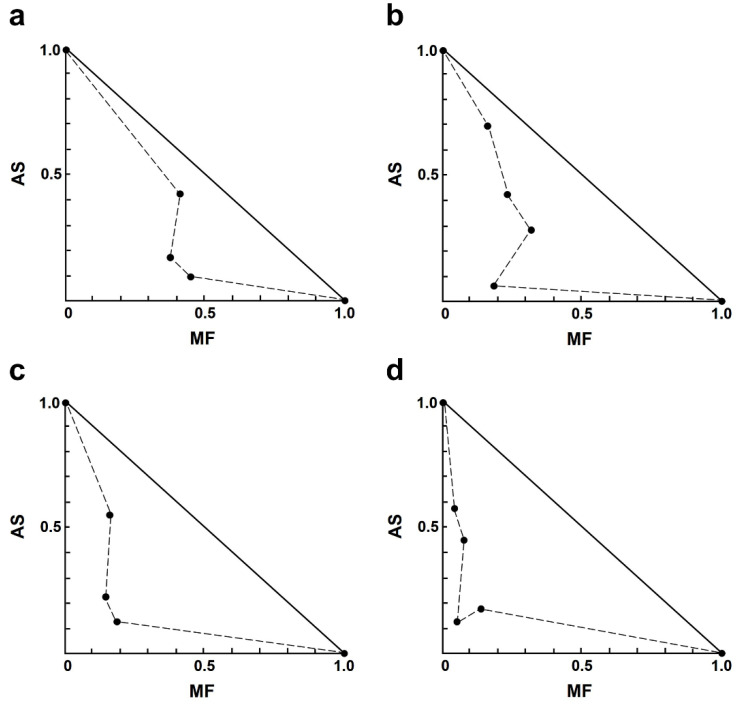
Representative normalized isobologram of the interaction between AS and MF on *L. infantum* intracellular amastigotes. The *X*-and *Y*-axis represent dose-normalized isobolograms for AS and MF, normalization of the dose with IC50 to unity on both axes. (**a**) 1.0 µM AS plus 0.13, 0.25, and 0.50 µM MF. (**b**) 2.0 µM AS plus 0.06, 0.13, 0.25 and 0.50 µM MF. (**c**) 4.0 µM AS plus 0.13, 0.25, and 0.50 µM MF. (**d**) 8.0 µM AS plus 0.06, 0.13, 0.25, and 0.50 µM MF.

In the AS–MF combination, most of the combinations showed moderate to strong synergism (Table 3). Among the synergistic interactions of AS–MF, three combination concentrations exhibited moderate synergism, including 1.00 µM AS plus 0.13 µM MF, 2.00 µM AS plus 0.06 µM MF, and 4.00 µM AS plus 0.13 µM MF. Additionally, eight combinations of AS–MF demonstrated synergism, including 1.00 µM AS plus 0.25 or 0.50 µM MF, 2.00 µM AS plus 0.13 or 0.25 µM MF, 4.00 µM AS plus 0.25 or 0.5 µM MF, and 8.00 µM AS plus 0.06 or 0.13 or 0.50 µM MF. Moreover, two combinations of AS–MF showed strong synergism, including 2.00 µM AS plus 0.50 µM MF and 8.00 µM AS plus 0.25 µM MF. However, two combinations exhibited moderate antagonism and strong antagonism, 1.00 µM AS plus 0.06 µM MF and 4.00 µM AS plus 0.06 µM MF (Table 3). Isobologram analysis, representing the interaction between AS and MF, indicated synergy for 14 combinations with 14 data points falling below the line of additivity (Figure 2). Furthermore, combining 8.00 µM AS with 0.06 µM MF resulted in the highest dose reduction index (DRI) value, indicating a significant reduction in the dose of MF required, with a DRI value of 22.84.

**Figure 3:**
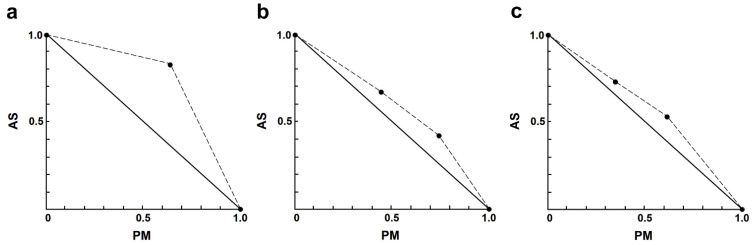
Representative normalized isobologram of the interaction between AS and PM on *L. infantum* intracellular amastigotes. The *X*-and *Y*-axis represent dose-normalized isobolograms for AS and PM, normalization of the dose with IC50 to unity on both axes. (**a**) 1.0 µM AS plus 35 µM PM. (**b**) 2.0 µM AS plus 35, and 70 µM PM. (**c**) 4.0 µM AS plus 35, and 70 µM PM.

In the AS–PM combination, the interactions ranged from slight to strong antagonism (Table 4). Only one combination exhibited nearly additive effects, which was 4.00 µM AS plus 35 µM PM. It is noteworthy that the interaction between AS and PM showed results (Figure 3) opposite to those observed in the AS–AmB or AS–MF combinations. 

### 2.4. Cytotoxicity of AS–AmB, AS–MF, and AS–PM Combinations to THP-1-Derived Macrophages

**Figure 4:**
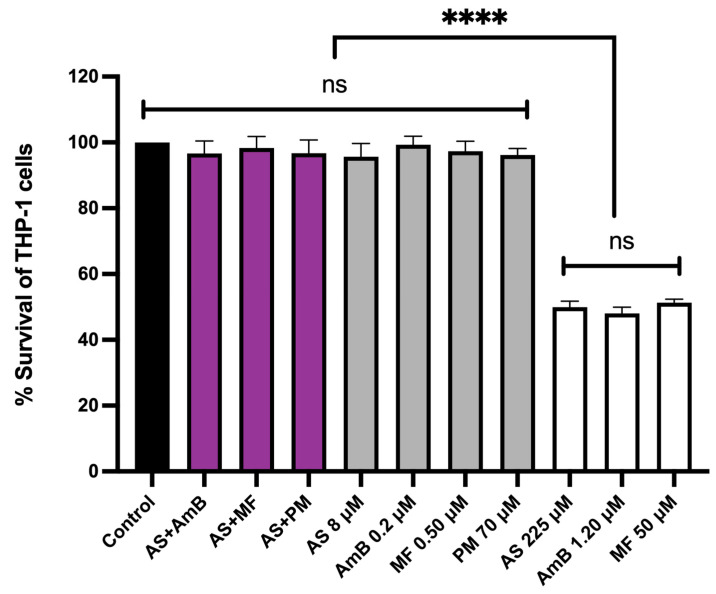
Cytotoxicity of AS in combination with AmB or MF or PM on THP-1-derived macrophages. The *X*-axis represents the different concentrations of drugs (µM). The black bar represents the untreated control; purple bars represent THP-1 cells treated with 8 µM AS + 0.2 µM AmB, 8 µM AS + 0.5 µM MF and 8 µM AS + 70 µM PM; gray bars represent THP-1 cells treated with 8.00 µM AS, 0.20 µM AmB, 0.5 µM MF and 70 µM PM; white bars represent THP-1 cells treated the drugs with near or equal to their CC_50_ value (225 µM AS, 1.20 µM AmB, and 50 µM MF). Statistical differences between the effects of AS, AmB, or MF at their respective CC_50_ values and their combinations or the untreated control are indicated as follows: ns indicates no significant difference, **** indicates a *p*-value of ≤0.0001.

The cytotoxicity of the combinations, 8.00 µM AS + 0.20 µM AmB and 8.00 µM AS + 0.50 µM MF, at their highest synergistic concentrations was evaluated in THP-1-derived macrophages. The cytotoxicity of these combinations was compared to the individual effects of 8.00 µM AS, 0.20 µM AmB, 0.5 µM MF, and 70 µM PM, as well as the CC_50_ concentrations of AS, AmB, and MF that were prepared and used as controls. Interestingly, the highest synergistic concentrations of both combinations, 8.00 µM AS + 0.20 µM AmB and 8.00 µM AS + 0.50 µM MF, did not exhibit any cytotoxic effects on the THP-1 cells when compared to the untreated control (Figure 4).

## 3. Discussion

This study investigated the interaction between AS and three antileishmanial drugs currently used in the treatment of *L. infantum*, the primary causative agent of zoonotic visceral leishmaniasis. The goal was to reduce the required doses of antileishmanial drugs and their associated toxicity. Initially, the individual effects of AS, AmB, MF, and PM on both the promastigote and intracellular amastigote stages of *L. infantum* were evaluated to determine their IC_50_ values. The cytotoxicity of these drugs to THP-1-derived macrophage was also assessed to establish CC_50_ values for each drug. 

The results revealed that AS exhibited higher efficacy against intracellular amastigotes (IC_50_ = 5.88 µM) compared to promastigotes (IC_50_ = 121.43 µM), consistent with previous findings in *L*. *martiniquensis* [35]. Artesunate (AS) is one of the artemisinin derivatives (ARTs). Previously, ARTs have been reported to exert both direct and indirect effects on *Leishmania* parasites [31], including inducing oxidative stress through free radical production and triggering cell-cycle arrest and apoptosis [28,36]. They also activate host cells to generate reactive oxygen species (ROS), leading to mitochondrial damage and altered of nitric oxide (NO) production in host macrophages [37]. 

MF and PM showed greater potency against the intracellular amastigotes than the extracellular promastigotes, with IC_50_ values of 0.37 µM and 64.30 µM, respectively. For AmB, the IC_50_ increased in the amastigotes (IC_50_ = 0.14 µM) compared to the promastigotes (IC_50_ = 0.02 µM). The differential susceptibility of the amastigotes and promastigotes to AS, AmB, MF, and PM may be attributed to distinct metabolic profiles and virulence gene expression in each stage [38]. 

The combination study between AS and AmB involved treating intracellular amastigotes with 16 different concentrations of AS–AmB combinations. The effects of the drug combinations were assessed using parasite rescue and transformation assays, and the interaction between two drugs was calculated using the Chou–Talalay combination index (CI) [39]. Our results demonstrated synergistic effects in 12 combinations of AS–AmB, with the best dose reduction observed at the concentration of 8.00 µM AS + 0.03 µM AmB, which achieved an approximately 9.20-fold reduction in the required AmB dosage (Table 2). The synergistic interaction between AS and AmB may be attributed to AS enhancing the ability of AmB to bind with ergosterol on the parasite surface membrane, disrupting parasite membrane function. There have been reports of interactions between artemisinins and AmB in other *Leishmania* species. For instance, Rahaman et al. [40] showed additive and synergistic effects of artemisinin and AmB in *L*. *donovani* promastigotes, while Intakhan et al. [35] demonstrated strong synergistic effects between AS and AmB on the intracellular amastigotes of the parasite *L*. *martiniquensis*. Although the synergistic interaction mechanism between artemisinins and AmB in *Leishmania* remains unexplored, a study on the pathogenic yeast *Candida albicans* revealed a synergistic interaction between artemisinin and AmB [41]. This report revealed that artemisinin upregulated the gene expression of the ergosterol biosynthesis pathway and enhanced ergosterol content on the yeast surface membrane, thereby increasing AmB binding to membrane ergosterol. As both *Leishmania* parasites and fungi use ergosterol as their primary membrane sterol, AmB is expected to bind to the cell membrane in a similar manner as in yeast, permeabilizing the parasite lipid bilayers to ion exchanges [42]. This process leads to potassium ion leakage, resulting in toxic effects due to the absence of intracellular ionic substances and the induction of ROS-based oxidative damage. Based on our findings, the synergistic effects of AS with AmB against the intracellular amastigotes of *L. infantum* could be explained by AS enhancing the ability of AmB to bind with ergosterol on the parasite surface membrane, thereby disrupting parasite membrane function. However, the exact mechanism in *Leishmania* remains unclear. Thus, further investigation on the mechanism of ergosterol level increase in *Leishmania* is required. 

In the case of the AS–MF combination, a majority of the combinations demonstrated synergism ranging from moderate to strong. The combinations of 8.00 µM AS + 0.06 µM MF and 8.00 µM AS + 0.25 µM MF resulted in a significant dose reduction in MF by approximately 22.84 and 18.97 times, respectively (Table 3). The underlying mechanisms of this synergistic interaction remain to be elucidated. Rahaman et al. [40] showed the additive and synergistic effects when artemisinin was combined with MF in *L*. *donovani* promastigotes, possibly due to targeting distinct mechanisms within the parasite, thereby inducing parasitic cell death. Miltefosine (MF), an alkyl-lysophospholipid analog, has been shown to exhibit anti-tumor and anti-microbial activities [43]. It disrupts lipid metabolism as well as phospholipid and sterol biosynthesis, while also impairing the oxygen consumption rate and ATP levels by inhibiting mitochondrial cytochrome C oxidase [44]. Furthermore, it can induce apoptosis [45]. Recent studies suggested that miltefosine might also influence sphingosine-activated Ca^2+^ channels and has a direct effect on acidocalcisomes [46]. In *Leishmania*, MF inhibits phosphatidylcholine biosynthesis, leading to a decrease in phosphatidylcholine concentrations but an increase in phosphatidylethanolamine levels [47]. Additionally, MF decreases fatty acid and diacylglycerol levels while elevating triglyceride concentrations. Some previous studies have suggested an increase in ergosterol levels [47,48]. It also has been reported that MF interacts with membrane ergosterol and integrates into the cell membrane [49,50]. These findings suggest that AS could elevate the level of ergosterol, a binding target of MF, thus enhancing the susceptibility of the parasite to the AS–MF combination. However, future studies should investigate the binding of MF to *Leishmania* ergosterol upon exposure to AS.

The results of the interaction study between AS and PM demonstrate contrasting outcomes compared to the AS–AmB and AS–MF combinations. The majority of the tested AS–PM concentration combinations displayed antagonistic effects, ranging from slight antagonism to strong antagonism. These findings suggest caution regarding the combined use of AS and PM for leishmaniasis treatment and emphasize the importance of in vitro drug interaction examinations before initiating in vivo studies or clinical trials. Currently, no studies have elucidated the interaction between AS and PM in *Leishmania* or in other microorganisms. Investigations into paromomycin-resistant lines indicate that the primary target of PM is the translation machinery in *Leishmania*, as evidenced by the proteomic analyses of susceptible and resistant *L*. *donovani* lines and the structural analyses of the *Leishmania* ribosome in complex with the drug [51,52,53].

Our study has revealed promising results regarding the combinations of AS–AmB and AS–MF, showing synergism and strong synergism, respectively. These combinations have demonstrated a significant reduction in the required doses of AmB (by 9.20 times) and MF (by 22.84 times). Moreover, the combination of AS–AmB and AS–MF showed no cytotoxicity to THP-1-derived macrophages at the highest concentration used in combination, suggesting that AS–AmB and AS–MF combinations have a high safety index values for chemotherapy in the treatment of VL. However, further investigation is necessary to assess efficacy, toxicity, and safety of these combinations in laboratory animals before proceeding to clinical trials. 

Previous investigations have reported the effectiveness of ARTs in treating murine leishmaniasis models. Studies have shown that artemisinin and artemether effectively control parasites and reduce cutaneous lesions in *L*. *major*-infected BalB/c mice [54,55]. In murine visceral leishmaniasis, artemisinin has demonstrated high efficacy in treating VL [30] and reducing hepatosplenomegaly [28]. Artesunate (AS) has shown particularly promising results in alleviating tissue inflammation and neuroinflammation, thereby reducing pain and discomfort in *L*. *amazonensis*-infected mice [56]. Combination therapies involving AS and diminazene have effectively reduced parasite load in the spleen of *L*. *donovani*-infected BalB/c mice [57]. Furthermore, AS has successfully treated canine leishmaniasis without side effects, implying its potential for human applications [32].

However, there are currently no in vivo studies examining the effects of AS–AmB or AS–MF combinations on *Leishmania*. Therefore, further investigation is needed to evaluate the in vivo efficacy of AS in combination with AmB or MF against *L. infantum*-infected laboratory animals. Such investigations may lead to the development of novel antileishmanial drug regimens that are highly efficacious, low toxicity, and reduce the emergence of drug resistance. This would ultimately result in improved patient outcomes by minimizing adverse effects, reducing hospitalization time, and decreasing the risk of opportunistic infections in hospital settings.

In conclusion, the combination of AS–AmB and AS–MF have demonstrated promising synergistic interactions against intracellular amastigotes of *L. infantum*, with potential mechanisms of synergy proposed. However, caution is advised regarding the AS–PM combination due to antagonistic interactions. This study underscores the importance of conducting drug interaction studies to develop effective therapeutic strategies that improve efficacy, reduce toxicity, prevent drug resistance in parasites, and enhance patient treatment outcomes.

## 4. Materials and Methods

### 4.1. Drugs

The following four commercially available drugs were used in this study: Artesunate (AS; Sigma-Aldrich, St. Louis, MO, USA), paromomycin (PM; Sigma-Aldrich, St. Louis, MO, USA), Miltefosine (MF; Sigma-Aldrich, St. Louis, MO, USA), and amphotericin B deoxycholate 250 µg/mL (AmB; Gibco, Grand Island, NY, USA). AS was prepared as a 20 mM stock solution in dimethyl sulfoxide (DMSO; Sigma-Aldrich, St. Louis, MO, USA), while MF and PM were prepared as 25 mM and 100 mM stock solutions, respectively, in sterile deionized water. All stock solutions were stored at −20 °C until use. Prior to each experiment, fresh dilutions of AS, AmB, MF, and PM were prepared in the culture medium and used immediately.

### 4.2. Parasite Strain

The *Leishmania infantum* (syn. *chagasi*) (MHOM/BR/74/PP75) strain, obtained from the American Type Culture Collection (ATCC-50133), was used in this study. Promastigotes were routinely cultured in Schneider’s Insect Medium (SIM) supplemented with 10% heat-inactivated fetal bovine serum (FBS; Gibco, Grand Island, NY, USA) and 25 µg/mL gentamicin sulfate (Sigma-Aldrich, St. Louis, MO, USA). The culture medium was maintained at pH 6.8 and a temperature of 26 °C. To ensure parasite viability, the promastigotes were sub-cultured into fresh medium every four days.

### 4.3. THP-1 Cell Culture 

The human monocytic cell line (THP-1 cell), obtained from the American Type Culture Collection (ATCC-TIB-202), was cultured in RPMI-1640 medium (pH 7.4; Gibco, Grand Island, NY, USA) supplemented with 10% (*v*/*v*) fetal bovine serum (FBS) and maintained at 37 °C in a 5% CO_2_ environment. To preserve the differentiation capacity of the cells, sub-passaging was performed every 3–4 days, ensuring that the cell density did not exceed 1 × 10^6^ cells/mL. To prepare THP-1-derived macrophages, The cells (2.5 × 10^5^ cells/mL) were treated with phorbol 12-myristate 13-acetate (PMA; Sigma-Aldrich, St. Louis, MO, USA) at a final concentration of 10 ng/mL [58]. Subsequently, 100 μL of the PMA-treated cells were dispensed into each well of a 96-well culture plate and incubated at 37 °C with 5% CO_2_ for 24 h. Following this incubation period, cells were washed with pre-warmed RPMI-1640 medium to remove residual PMA, and the medium was replaced with fresh RPMI-1640 supplemented with 10% (*v*/*v*) FBS. The cells were then incubated at 37 °C with 5% CO_2_ for an additional 72 h. After this period, cells were washed again with pre-warmed RPMI-1640 medium and prepared for use in cytotoxicity assays or in vitro amastigote assays.

### 4.4. Promastigote Assay 

The antileishmanial activity of AS, AmB, MF, and PM against *L. infantum* promastigotes was evaluated using the alamarBlue™ cell viability assay (Thermo Fisher Scientific, Waltham, MA, USA), with a slightly modified protocol based on Intakhan et al. [35]. Briefly, promastigotes in the logarithmic phase (Day 3) of *L. infantum* were adjusted to a concentration of 2 × 10^6^ cells/mL in SIM, pH 6.8, supplemented with 10% fetal bovine serum (FBS) and 25 µg/mL gentamicin sulfate. Next, 50 µL of the promastigote suspension was added to each well of 96-well culture plates (Nunc, Roskilde, Denmark). Promastigotes were then treated with various concentrations of 3.91–500 µM AS, 0.004–0.50 µM AmB, 1.56–100 µM MF, 15.63–1000 µM PM, or the culture medium alone (control), with 50 µL per well, resulting in a final volume of 100 µL per well. The plates were incubated at 26 °C for 72 h. After a 48 h exposure period to the drugs, 10 μL of alamarBlue™ cell viability reagent (10% *v*/*v*) was added to each well, followed by an additional 24 h incubation at 26 °C. The proliferation of promastigotes was measured using a Synergy Mx™ microplate spectrophotometer (BioTek, Winooski, VT, USA) at 540 nm and 590 nm wavelengths. Control wells containing only culture medium and the alamarBlue™ reagent (10% *v*/*v*) were included as controls. The IC_50_ value, representing the drug concentration required to inhibit 50% of promastigote growth, was determined by generating a concentration–response curve using GraphPad Prism software version 9 (GraphPad Software Inc., San Diego, CA, USA). These assays were replicated three times, with each experiment conducted in duplicate. The results are presented as the mean ± standard deviation.

### 4.5. Cytotoxicity Assay

The cytotoxicity of AS, AmB, MF, and PM was assessed in THP-1-derived macrophages. The cells were treated to different concentrations of 7.81–1000 µM AS, 0.16–15 µM AmB, 3.91–250 µM MF, and 156.25–20,000 µM PM, or medium alone (control), and incubated for 72 h at 37 °C, 5% CO_2_. After 68 h of drug exposure, 10 μL of alamarBlue™ reagent was added to each well, and the incubation was extended for an additional 4 h at 37 °C with 5% CO_2_. Cell viability was determined using a Synergy Mx™ microplate spectrophotometer (BioTek, Winooski, VT, USA) at 540 and 590 nm. Control wells containing culture medium and alamarBlue™ reagent (10% *v*/*v*) were also included. The CC_50_ value, defined as the drug concentration required to induce 50% cell death, was determined using a concentration–response curve generated with GraphPad Prism software version 9 (GraphPad Software Inc., San Diego, CA, USA). These assays were conducted in three independent experiments, with duplicates within each experiment. The selectivity index (SI) values of AS, AmB, MF, and PM were calculated by dividing the CC_50_ for macrophages by the IC_50_ for parasites (intracellular amastigotes). A drug with an SI value higher than 10 was considered effective and safe [34].

### 4.6. Preparation of Promastigotes to Infect THP-1-Derived Macrophages

*Leishmania infantum* promastigotes in the late logarithmic phase (day 4) were subcultured into acidic SIM medium, pH 5.5, supplemented with 20% FBS and 25 µg/mL gentamicin sulfate, to induce metacyclogenesis [59]. Cultures were then incubated for 5 days at 26 °C. Metacyclogenesis is the process by which promastigotes transform into infective metacyclic promastigotes. To ensure the presence of sufficient number of metacyclic promastigotes, stationary phase promastigote cultures were used. These cultures contained more than 70% metacyclic promastigotes. The identification of metacyclic promastigotes was based on specific morphological features, including body width, body length, and flagella length, as described by Rogers et al. [60]. Metacyclic promastigotes have a body length of less than 8 μm, a body width of 1 μm, and a flagellum length greater than the body length. The resulting metacyclic promastigotes were then used to infect THP-1 macrophages. The metacyclic promastigotes, being the infective form of the parasite, establish infection within the macrophages and continue their life cycle.

### 4.7. Intracellular Amastigote Assay and Parasite Rescue and Transformation Assay

To evaluate the effects of AS, AmB, MF, and PM on intracellular amastigotes of *L. infantum*, the infected cells were prepared as follows. THP-1-derived macrophages were infected with stationary phase promastigotes of *L. infantum* suspended in RPMI-1640 medium supplemented with 2% FBS for 24 h, at a parasite/macrophage ratio of 10:1. Subsequently, the infected cells were washed with pre-warmed RPMI-1640 medium to remove non-internalized promastigotes, and the medium was replaced with RPMI-1640 medium (supplemented with 2% FBS) containing different concentrations of 0.78–100 µM AS, 0.006–0.8 µM AmB, 0.03–4.00 µM MF, and 7.5–960 µM PM, and incubated for 48 h at 37 °C in 5% CO_2_ environment. 

After the 48 h exposure to the drugs, the survival of amastigotes within the macrophages was assessed using a parasite rescue and transformation assay, as described by Intakhan et al. [35]. Briefly, the cells were washed with serum-free RPMI-1640 and then added 20 μL/well of RPMI-1640 containing 0.05% sodium dodecyl sulfate (SDS) (*w*/*v*) for 30 s, to disrupt the cell membrane and release intracellular amastigotes. The SDS was neutralized by adding 180 μL/well of SIM, pH 6.8, supplemented with 20% FBS, and the cells were incubated 26 °C for 96 h. Under these conditions, the released intracellular amastigotes transformed into promastigotes and initiated proliferation. After 96 h of incubation, 20 µL of alamarBlue™ reagent was added to each well and incubated at 26 °C for an additional 24 h. The proliferation of promastigotes was measured using a Synergy Mx™ microplate spectrophotometer (BioTek, Winooski, VT, USA) at wavelengths of 540 nm and 590 nm. Control wells with non-infected macrophages treated with 0.05% SDS for 30 s (Control lysis) and wells containing alamarBlue™ reagent (10% *v*/*v*) in culture medium were included as controls. The IC_50_ value, representing the drug concentration required to inhibit 50% of promastigote growth, was determined using GraphPad Prism software version 9 from three independent experiments. The results were expressed as mean ± standard deviation.

### 4.8. Drug Combination Assay on Intracellular Amastigotes

To determine the combination effects of AS with AmB or MF and PM against intracellular amastigotes, checkerboard assays were performed. AS, AmB, MF, and PM were prepared at concentrations near or equal to their IC_50_ values. These drugs were serially two-fold diluted in RPMI-1640 medium supplemented with 2% FBS and 25 µg/mL gentamicin sulfate. The concentrations used were as follows: AS: 1, 2, 4, and 8 µM, AmB: 0.03, 0.05, 0.10 and 0.20 µM, MF: 0.06, 0.13, 0.25, 0.50 µM, and PM: 8.75, 17.5, 35, 70 µM. Each drug was combined with different dilutions, resulting in a matrix of 16 different combinations for AS–AmB, AS–MF, and AS–PM, as shown in Table 2. 

*L. infantum*-infected THP-1 macrophages were prepared in 96-well plates with a parasite/macrophage ratio of 10:1, as described previously. After 24 h of infection, the infected macrophages were washed to remove non-internalized parasites and then treated with AS, AmB, MF, PM alone, or AS–AmB, AS–MF, AS–PM combinations for 48 h at 37 °C in a 5% CO_2_ environment. After the 48 h drug exposure, the parasite rescue and transformation assays were performed as described earlier. The percentage of growth inhibition for each combination concentration was calculated by comparing it to the untreated control. The results were expressed as the mean ± standard deviation of three independent experiments. 

**Figure 5:**
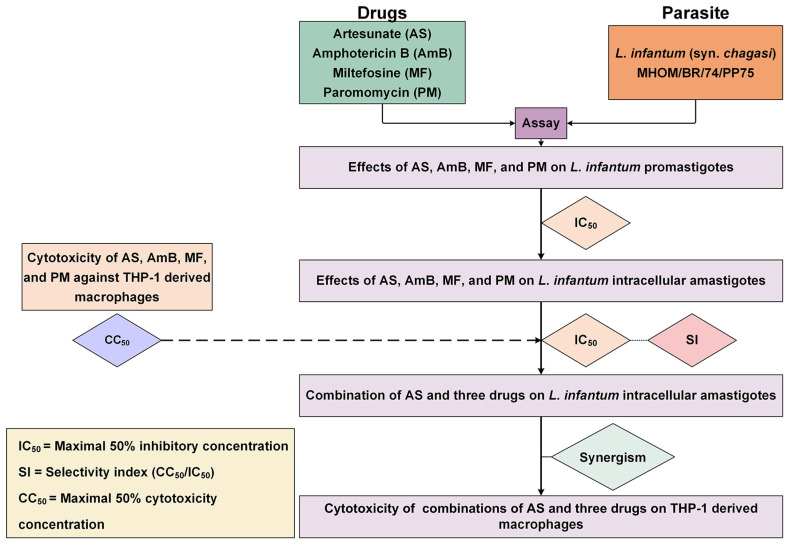
Overall study of the interaction between AS and three antileishmanial drugs against *L. infantum*. The diagram illustrates the process used for evaluating the activity of drugs against the parasite, and THP-1-derived macrophages and investigating drug interaction and cytotoxicity of the drug combinations.

The interactions between AS and the three antileishmanial drugs were analyzed using the Chou–Talalay combination index method using CompuSyn software version 1.0.1 (ComboSyn Inc., Paramus, NJ, USA). The term ‘combination index’ is used for the quantification of synergism or antagonism between two drugs. The CI value is calculated based on the multiple drug–effect equation. If the sum of these two fractional terms equals 1, additivity is indicated. If the CI value is less than 1, synergism is indicated, and if the CI value is greater than 1, antagonism is indicated [39]. The results of AS–AmB, AS–MF, and AS–PM interactions were classified based on the CI value, according to Chanmol et al. [61]. The dose reduction index (DRI) was calculated to determine the fold reduction in dose when drugs were used in combination compared to the dose of each drug used alone. Isobolograms were analyzed using CompuSyn software (ComboSyn Inc., Paramus, NJ, USA) to provide a graphical presentation of the two drug interactions. A point below, on, or above the line of additivity indicated synergy, additivity, or antagonism, respectively. The overall study is illustrated in Figure 5.

### 4.9. Statistical Analysis

Statistical analysis was performed by using GraphPad Prism software version 9 (Graphpad Software Inc., San Diego, CA, USA). The mean and standard deviation (SD) were calculated based on data obtained from three independent experiments. To assess the statistical differences in growth inhibition between the drug combinations and AS alone, a one-way analysis of variance (ANOVA) was performed, followed by Dunnett’s multiple comparison tests using the same software. For evaluating the statistical differences in cytotoxicity among the drug combinations, a one-way ANOVA was used, followed by Bonferroni’s multiple comparison tests. Statistical significance was determined for *p*-values < 0.05.

## Figures and Tables

**Table 1:** Antileishmanial activity of AS, AmB, MF, and PM against *L. infantum* promastigotes and intracellular amastigotes. The selectivity index of those drugs also calculated from 50% inhibitory concentration and cytotoxicity (CC_50_) were shown in this table.

Drugs (µM)	IC_50_ ^5^ (Promastigote)	IC_50_ (Amastigote)	CC_50_ ^6^	SI ^7^ (Amastigote)
AS ^1^	121.43 ± 2.05	5.88 ± 1.67	225.77 ± 21.71	38.40
AmB ^2^	0.02 ± 0.002	0.14 ± 0.02	1.20 ± 0.27	8.57
MF ^3^	2.95 ± 0.18	0.37 ± 0.11	51.12 ± 0.65	138.16
PM ^4^	114.93 ± 9.46	64.30 ± 2.37	>20,000	>311

^1^ AS: Artesunate. ^2^ AmB: Amphotericin B deoxycholate. ^3^ MF: Miltefosine. ^4^ PM: Paromomycin. ^5^ IC_50_: half-maximal inhibitory concentration. ^6^ CC_50_: half-maximal cytotoxic concentration. ^7^ SI: selectivity index value (CC_50_/IC_50_).

**Table 2:** Effects of AS and AmB combinations on intracellular amastigotes of *L. infantum*.

Drug Combination Non-Constant Ratio (µM) ^1^			Growth Inhibition (%) ^2^	CI ^3^	Interaction	Dose Reduction Index (DRI) ^4^	
Combinations	AS	AmB				AS	AmB
1	1.00	0.03	49.1 ± 3.87 **	0.68	synergism	2.04	5.39
2	1.00	0.05	63.36 ± 2.27 ****	0.55	synergism	3.45	3.85
3	1.00	0.10	86.3 ± 4.35 ****	0.44	synergism	11.19	2.84
4	1.00	0.20	87.85 ± 5.14 ****	0.76	slight synergism	12.65	1.48
5	2.00	0.03	5.07 ± 4.39 ****	14.06	very strong antagonism	0.07	2.26
6	2.00	0.05	62.23 ± 2.76 ***	0.87	slight synergism	1.65	3.80
7	2.00	0.10	84.79 ± 4.40 ****	0.57	synergism	4.99	2.74
8	2.00	0.20	89.21 ± 2.27 ****	0.79	moderate synergism	7.14	1.54
9	4.00	0.03	30.51 ± 3.21 ****	4.26	strong antagonism	0.25	4.26
10	4.00	0.05	81.53 ± 2.91 ***	0.69	synergism	2.02	5.10
11	4.00	0.10	89.87 ± 2.97 ****	0.58	synergism	3.81	3.14
12	4.00	0.20	94.47 ± 3.72 ****	0.67	synergism	6.90	1.91
13	8.00	0.03	85.19 ± 1.59	0.89	slight synergism	1.28	9.20
14	8.00	0.05	84.83 ± 1.30	0.98	nearly additive	1.25	5.84
15	8.00	0.10	86.14 ± 3.20	1.08	nearly additive	1.38	2.83
16	8.00	0.20	96.83 ± 1.62 ***	0.61	synergism	5.85	2.28

^1^ Concentration (µM) of AS combined with AmB. ^2^ Growth inhibition (%) (mean ± SD) obtained from antileishmanial activities against *L. infantum* intracellular amastigotes using AS or AmB alone and their combinations. ^3^ Combination index (CI) values indicate the levels of drug interaction. CI of less than 0.10 = very strong synergism, CI 0.10–0.30 = strong synergism, CI 0.30–0.70 = synergism, CI 0.70–0.85 = moderate synergism, CI 0.85–0.90 = slight synergism, CI 0.90–1.10 = nearly additive, CI 1.10–1.20 = slight antagonism, CI 1.20–1.45 = moderate antagonism, CI 1.45–3.30 = antagonism, CI 3.30–10.00 = strong antagonism, CI of more than 10.00 = very strong antagonism. ^4^ Dose reduction index (DRI), a measurement of how much drug dosage can be reduced in a combination while maintaining the same degree of effect compared to the dosage of drug used individually. Statistical differences between the effects of AS alone and its combination with AmB are represented by the following: ** *p* ≤ 0.01, *** *p* ≤ 0.001, **** *p* ≤ 0.0001.

**Table 3:** Effects of AS and MF combinations on intracellular amastigotes of *L. infantum*.

Drug Combination Non-Constant Ratio (µM) ^1^			Growth Inhibition (%) ^2^	CI ^3^	Interaction	Dose Reduction Index (DRI) ^4^	
Combinations	AS	MF				AS	MF
1	1.00	0.06	25.75 ± 2.64	1.44	moderate antagonism	0.96	2.52
2	1.00	0.13	51.15 ± 4.18 ****	0.84	moderate synergism	2.35	2.42
3	1.00	0.25	75.99 ± 2.55 ****	0.55	synergism	5.72	2.63
4	1.00	0.50	87.42 ± 2.33 ****	0.54	synergism	10.77	2.22
5	2.00	0.06	57.28 ± 1.70 **	0.86	moderate synergism	1.43	6.19
6	2.00	0.13	71.17 ± 5.26 ****	0.66	synergism	2.34	4.29
7	2.00	0.25	80.36 ± 5.46 ****	0.60	synergism	3.52	3.12
8	2.00	0.50	96.36 ± 5.51 ****	0.25	strong synergism	15.81	5.41
9	4.00	0.06	27.86 ± 3.67 ****	4.18	strong antagonism	0.26	2.79
10	4.00	0.13	80.84 ± 4.83 **	0.72	moderate synergism	1.80	6.13
11	4.00	0.25	93.08 ± 4.75 ****	0.36	synergism	4.58	6.90
12	4.00	0.50	96.25 ± 4.71 ****	0.32	synergism	7.71	5.30
13	8.00	0.06	90.50 ± 3.06 *	0.62	synergism	1.74	22.84
14	8.00	0.13	92.83 ± 3.13 **	0.53	synergism	2.22	12.93
15	8.00	0.25	98.40 ± 1.60 ***	0.18	strong synergism	7.79	18.97
16	8.00	0.50	97.59 ± 3.07 ***	0.32	synergism	5.57	7.18

^1^ Concentration (µM) of AS combined with MF. ^2^ Growth inhibition (%) (mean ± SD) obtained from antileishmanial activities against *L. infantum* intracellular amastigotes using AS or MF alone and their combinations. ^3^ Combination index (CI) values indicate the levels of drug interaction. CI of less than 0.10 = very strong synergism, CI 0.10–0.30 = strong synergism, CI 0.30–0.70 = synergism, CI 0.70–0.85 = moderate synergism, CI 0.85–0.90 = slight synergism, CI 0.90–1.10 = nearly additive, CI 1.10–1.20 = slight antagonism, CI 1.20–1.45 = moderate antagonism, CI 1.45–3.30 = antagonism, CI 3.30–10.00 = strong antagonism, CI of more than 10.00 = very strong antagonism. ^4^ Dose reduction index (DRI), a measurement of how much drug dosage can be reduced in a combination while maintaining the same degree of effect compared to the dosage of drug used individually. Statistical differences between the effects of AS alone and its combination with MF are represented by the following: * *p* ≤ 0.05, ** *p* ≤ 0.01, *** *p* ≤ 0.001, **** *p* ≤ 0.0001.

**Table 4:** Effects of AS and PM combinations on intracellular amastigotes of *L. infantum*.

Drug Combination Non-Constant Ratio (µM) ^1^			Growth Inhibition (%) ^2^	CI ^3^	Interaction	Dose Reduction Index (DRI) ^4^	
Combinations	AS	PM				AS	PM
1	1.00	8.75	4.37 ± 4.79 ****	7.70	strong antagonism	0.14	2.63
2	1.00	17.50	12.03 ± 3.21 ***	3.19	strong antagonism	0.37	1.96
3	1.00	35.00	32.98 ± 4.09	1.47	antagonism	1.20	1.56
4	1.00	70.00	46.15 ± 3.03 **	1.55	antagonism	2.00	0.95
5	2.00	8.75	25.93 ± 5.44 **	2.45	antagonism	0.44	5.52
6	2.00	17.50	31.78 ± 4.73 *	2.07	antagonism	0.57	3.06
7	2.00	35.00	57.06 ± 3.99 **	1.11	slight antagonism	1.50	2.24
8	2.00	70.00	68.63 ± 3.90 ****	1.17	slight antagonism	2.36	1.34
9	4.00	8.75	24.73 ± 5.08 ****	4.99	strong antagonism	0.21	5.39
10	4.00	17.50	54.13 ± 3.16 *	1.72	antagonism	0.67	4.29
11	4.00	35.00	72.23 ± 3.90 *	1.07	nearly additive	1.38	2.86
12	4.00	70.00	78.47 ± 1.60 ***	1.15	slight antagonism	1.89	1.61
13	8.00	8.75	59.97 ± 3.84 ****	2.50	antagonism	0.42	9.36
14	8.00	17.50	78.12 ± 3.54	1.24	moderate antagonism	0.93	6.41
15	8.00	35.00	75.94 ± 1.28	1.54	antagonism	0.83	3.07
16	8.00	70.00	74.88 ± 1.13	1.94	antagonism	0.78	1.50

^1^ Concentration (µM) of AS combined with PM. ^2^ Growth inhibition (%) (mean ± SD) obtained from antileishmanial activities against *L. infantum* intracellular amastigotes using AS or PM alone and their combinations. ^3^ Combination index (CI) values indicate the levels of drug interaction. CI of less than 0.10 = very strong synergism, CI 0.10–0.30 = strong synergism, CI 0.30–0.70 = synergism, CI 0.70–0.85 = moderate synergism, CI 0.85–0.90 = slight synergism, CI 0.90–1.10 = nearly additive, CI 1.10–1.20 = slight antagonism, CI 1.20–1.45 = moderate antagonism, CI 1.45–3.30 = antagonism, CI 3.30–10.00 = strong antagonism, CI of more than 10.00 = very strong antagonism. ^4^ Dose reduction index (DRI), a measurement of how much drug dosage can be reduced in a combination while maintaining the same degree of effect compared to the dosage of drug used individually. Statistical differences between the effects of AS alone and its combination with PM are represented by the following: * *p* ≤ 0.05, ** *p* ≤ 0.01, *** *p* ≤ 0.001, **** *p* ≤ 0.0001.

## Data Availability

Data are contained within the article.

## Supplementary Materials

The following supporting information can be downloaded at: https://www.mdpi.com/article/10.3390/antibiotics13090806/s1, Figure S1: Concentration–response curves of AS, AmB, MF and PM against *L. infantum* (promastigotes and intracellular amastigotes) and THP-1-derived macrophages (cytotoxicity).
