# Supplementary material for: Synergistic Effects of Artesunate in Combination with Amphotericin B and Miltefosine against Leishmania infantum: Potential for Dose Reduction and Enhanced Therapeutic Strategies
Source: Antibiotics (Basel). 2024 Aug 26;13(9):806. doi: 10.3390/antibiotics13090806 (PMC11428804; doi:10.3390/antibiotics13090806)
Supplement: Supplementary file 1 [file antibiotics-13-00806-s001.zip › antibiotics-3140411-supplementary.pdf]

# Supplementary Materials

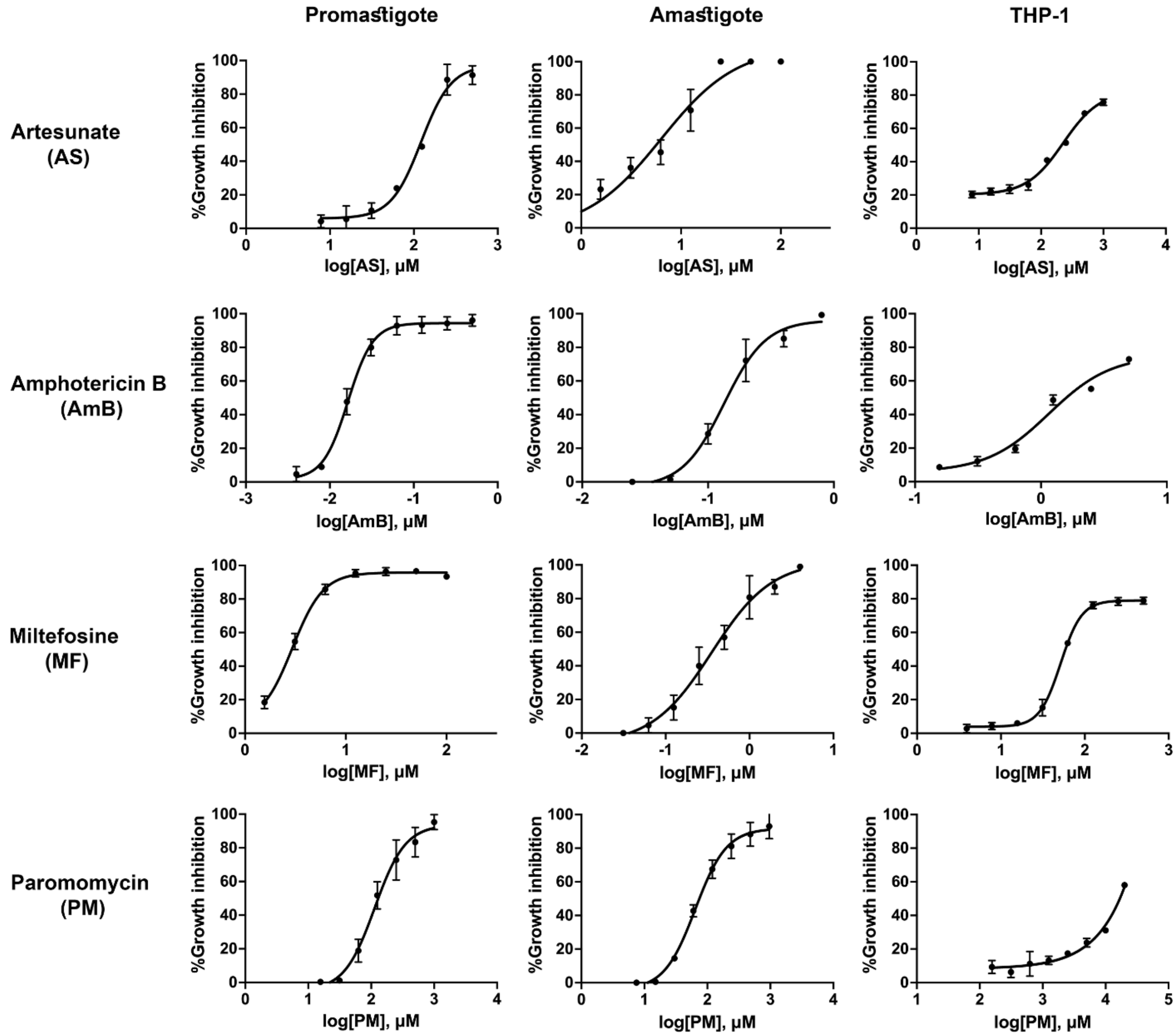


**Figure S1**. Concentration-response curves of AS, AmB, MF and PM against *L. infantum* (promastigotes and intracellular amastigotes) and THP-1 derived macrophages (cytotoxicity).
